# PFKFB3 regulates cancer stemness through the hippo pathway in small cell lung carcinoma

**DOI:** 10.1038/s41388-022-02391-x

**Published:** 2022-07-08

**Authors:** Prabhu Thirusangu, Upasana Ray, Sayantani Sarkar Bhattacharya, Derek B. Oien, Ling Jin, Julie Staub, Nagarajan Kannan, Julian R. Molina, Viji Shridhar

**Affiliations:** 1 Department of Experimental Pathology and Medicine, Mayo Clinic, Rochester, MN USA; 2 Division of Experimental Pathology, Department of Laboratory Medicine and Pathology, Center for Regenerative Medicine, Mayo Clinic, Rochester, MN USA; 3 Department of Medical Oncology, Mayo Clinic, Rochester, MN USA; 4 Present Address: Oncology R&D, AstraZeneca, Boston, MA USA

**Keywords:** Cell signalling, Cancer stem cells

## Abstract

PFKFB3 (6-phosphofructo-2-kinase) is the rate-limiting enzyme of glycolysis and is overexpressed in several human cancers that are associated with poor prognosis. High PFKFB3 expression in cancer stem cells promotes glycolysis and survival in the tumor microenvironment. Inhibition of PFKFB3 by the glycolytic inhibitor PFK158 and by shRNA stable knockdown in small cell lung carcinoma (SCLC) cell lines inhibited glycolysis, proliferation, spheroid formation, and the expression of cancer stem cell markers CD133, Aldh1, CD44, Sox2, and ABCG2. These factors are also associated with chemotherapy resistance. We found that PFK158 treatment and PFKFB3 knockdown enhanced the ABCG2-interacting drugs doxorubicin, etoposide, and 5-fluorouracil in reducing cell viability under conditions of enriched cancer stem cells (CSC). Additionally, PFKFB3 inhibition attenuated the invasion/migration of SCLC cells by downregulating YAP/TAZ signaling while increasing pLATS1 via activation of pMST1 and NF2 and by reducing the mesenchymal protein expression. PFKFB3 knockdown and PFK158 treatment in a H1048 SCLC cancer stem cell-enriched mouse xenograft model showed significant reduction in tumor growth and weight with reduced expression of cancer stem cell markers, ABCG2, and YAP/TAZ. Our findings identify that PFKFB3 is a novel target to regulate cancer stem cells and its associated therapeutic resistance markers YAP/TAZ and ABCG2 in SCLC models.

## Introduction

Small-cell lung cancer (SCLC) is an extremely malignant disease that often advances to treatment-resistant tumors. This cancer generally responds to initial treatment, but most patients will experience recurrence with an average survival of less than 6 months at this phase [1–4]. Resistance to conventional chemotherapies is an important clinical challenge. The presence of a small subset of cancer stem-like cells (CSC) that promote tumor initiation, expansion, epithelial-to-mesenchymal transition (EMT), metastasis, and relapse are associated with the development of resistant disease [5]. This CSC population has numerous crucial properties such as the ability to disproportionately replicate, differentiate and self-renew, in addition to having extensive inherent resistance to therapy. Although cytotoxic chemotherapy eradicates most tumor cells, CSCs are more protected and capable to recapture the heterogenic tumor mass through self-renewal with high expression of aldehyde dehydrogenase 1 A (Aldh1A), CD133, CD44 and Sox2 [5–8].

Dysregulation of the hippo pathway has been associated with cancer progression [9–11], including the abnormal expression and activity of yes-associated protein (YAP) and transcriptional co-activator with PDZ-binding motif (TAZ), and a deficiency in large tumor suppressor kinase 1/2 (LATS1/2) level. Recent evidence supports a role of YAP/TAZ in cancer stem cells and tumor recurrence [12, 13]. The CSC subpopulation can also efflux many chemotherapeutic agents through transporters as a mechanism of resistance to chemotherapy drugs [11, 14, 15]. CSCs show increased expression of ATP-binding cassette (ABC) transporters that reflect the efflux capacity and among the ABC transporter members, ATP-binding cassette sub-family G member 2 (ABCG2) has been shown to be associated with drug efflux and CSC formation [16–19]. We hypothesize that drugs targeting ABCG2 and stemness in SCLC will address the subpopulation of tumor cells that promote recurrence.

Numerous new therapies that target selective oncogenic signaling pathways in lung cancer have been developed and tested in clinical trials but have not yielded promising results. Hanahan and Weinberg’s recent update on the hallmarks of cancer [20] identified reprogramming of energy metabolism as playing a pivotal role in supporting the features of CSCs. CSCs are prone to maintain an abnormally high rate of aerobic glycolysis, a phenomenon known as the Warburg effect [21–23]. PFKFB3 (6-phosphofructo-2-kinase/fructose-2,6-bisphosphatase-3) is a key regulator of high glycolytic flux in cancers by catalyzing the synthesis of F2,6P2 which allosterically activates PFK-1, the rate-limiting enzyme of glycolysis [24–27]. PFKFB3 protein levels are overexpressed in a wide variety of cancers [25] and its expression or activity has been found to be strongly correlated with the aggressiveness and poor prognosis of the cancer [25, 28]. Thus, understanding the underlying mechanism by which CSCs acquire stemness properties through metabolic reprogramming can help to target CSCs. Recent evidence suggests inhibition PFKFB3 leads to apoptosis, cell cycle arrest, autophagy, DNA damage, and macropinocytosis [29–31]. However, the therapeutic efficacy of PFKFB3 inhibition targeting cancer stemness remain unidentified. In this study, we examined the effects of targeting PFKFB3 with genetic knockdown or with glycolytic inhibitor PFK158 (a specific inhibitor of PFKFB3) on cancer stemness, YAP/TAZ signaling, EMT, and therapeutic resistance for SCLC in vitro and in vivo. We found that targeting PFKFB3 offered a promising therapeutic approach for cancer therapeutics.

## Results

### PFKFB3 KD or inhibition by PFK158 attenuates stemness and induces apoptosis in CSC enriched SCLC cells

**Fig. 1:**
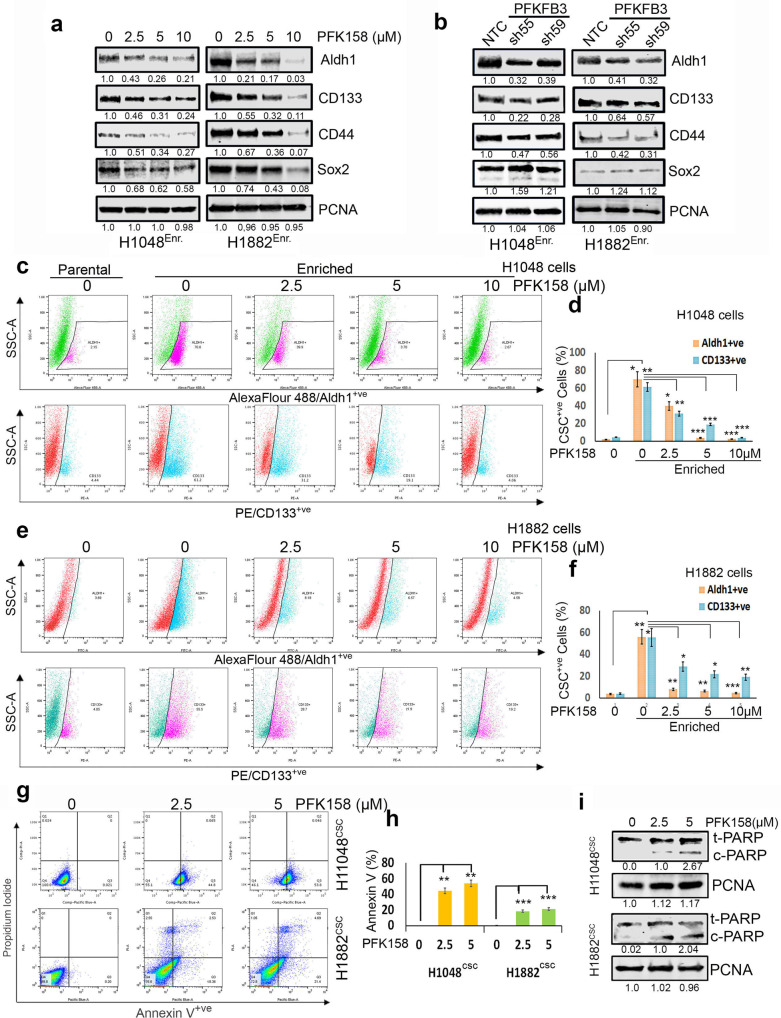
Deficient PFKFB3 causes downregulation of cancer stem cell markers and cell death. **a** Expression levels of CSC markers including Aldh1, CD133, CD44, and Sox2 in enriched 3D cultures of H1048 and H1882 cells by western blot analysis. **b** The effect of PFKFB3 depletion by shRNAs on CSC markers level in enriched 3D cultures of H1048 and H1882 cells. **c** Aldh1 (pink)/CD133 (blue) was very higher level in CSC enriched 3D culture compared to parental cells and exposure to PFK158 at indicated concentrations decreased detection of both Aldh1^+ve^/CD133^+ve^ levels in H1048 spheroids. The pink (first panel) and blue (second panel) gating boxed represent unique Aldh1^high^ /CD133^high^ cells in H1048 spheroids. **d** PFK158 mediated decrease of CSC markers Aldh1^+ve^/CD133^+ve^ cells. **e** Aldh1 (blue)/ CD133 (pink) expressing cells are more in 3D spheroids compared to parental 2D cultures and Dot plot for expression of cancer stemness markers Aldh1 and CD133 analyzed with FACS depicting concentration dependant decrease CSC markers upon PFK158 treatment. The blue (first panel) and pink (second panel) gating boxed represent unique Aldh1^high^ /CD133^high^ cells respectively, in H1882 spheroids. **f** Percentage of CSC Aldh1^+ve^/CD133^+ve^ cells in H1882 spheroids. **g** Determination of the early and late apoptotic cells after exposure to PFK158 at specified concentrations by Annexin V-Pacific Blue and PI dual stain in H1048^CSC^ and H1882^CSC^ spheroids. **h** Bar graph depicts percentage of Annexin-V-positive/apoptotic cells. **i** immunoblot analysis of cleaved PARP level after PFK158 treatment in H1048^CSC^ and H1882^CSC^ cells. PCNA used as loading control. The experiment was repeated thrice (*n* = 3), and the graphs are represented as mean ± S.D. Significance measured comparing individual groups with control is represented as *p < 0.05, **p < 0.01, ***p < 0.001.

In general, the expression levels of Aldh1, CD133, CD44, and Sox2 were significantly higher in CSC enriched 3D spheroids in enrichment medium compared to adherent cells in standard medium (Supplementary Fig. 1a). To determine if PFKFB3 expression impacts the cancer stem cells, we generated stable shRNA mediated PFKFB3 KD clones in H1048 and H1882 cells (sh55^PFKFB3^and sh59^PFKFB3^) along with non-targeted control (NTC) shRNA control cells. Efficient downregulation of PFKFB3 total and phosphorylated protein was verified in both H1048 and H1882 cells grown as spheroids (Supplementary Fig. 1b). Decreasing PFKFB3 by PFK158 (an inhibitor of PFKFB3) and genetic knockdown attenuated CSC marker expression in the enriched population for H1048 and H1882 cells (Fig. 1a–f). PFK158 significantly downregulated the expression of the four CSC markers in H1048 and H1882 cells in a dose-dependent manner (Fig. 1a). The expression of Aldh1, CD133, and CD44 were also considerably downregulated in H1048^shPFKFB3^ and H1882^shPFKFB3^ 3D spheroids, although no change in Sox2 expression was detected (Fig. 1b). The PFK158-induced decrease for Aldh1 and CD133 was verified by FACS analysis. The number of both Aldh1^+ve^ and CD133^+ve^ cells were substantially increased to 55-65% in enriched 3D-tumor spheres prior to drug treatment when compared to parental cells for H1048 and H1882 cell lines (Fig. 1c–f). In the CSC-enriched cells, PFK158 treatment decreased the amount of Aldh1^+ve^ and CD133^+ve^ cells. These data suggest that Aldh1^high^/CD133^high^ cells have a selective response to PFKFB3 inhibition. In the population of cells remaining after PFK158 treatment, we detected a significant increase in Annexin V positive cells (Fig. 1g, h), which suggests most of the remaining cells have a loss of membrane integrity and are in a stage of apoptosis. This was further verified by an increase in cleaved PARP after 2.5 and 5 µM PFK158 dosing (Fig. 1i).

### PFK158 inhibits proliferation of enriched SCLC cells

**Fig. 2:**
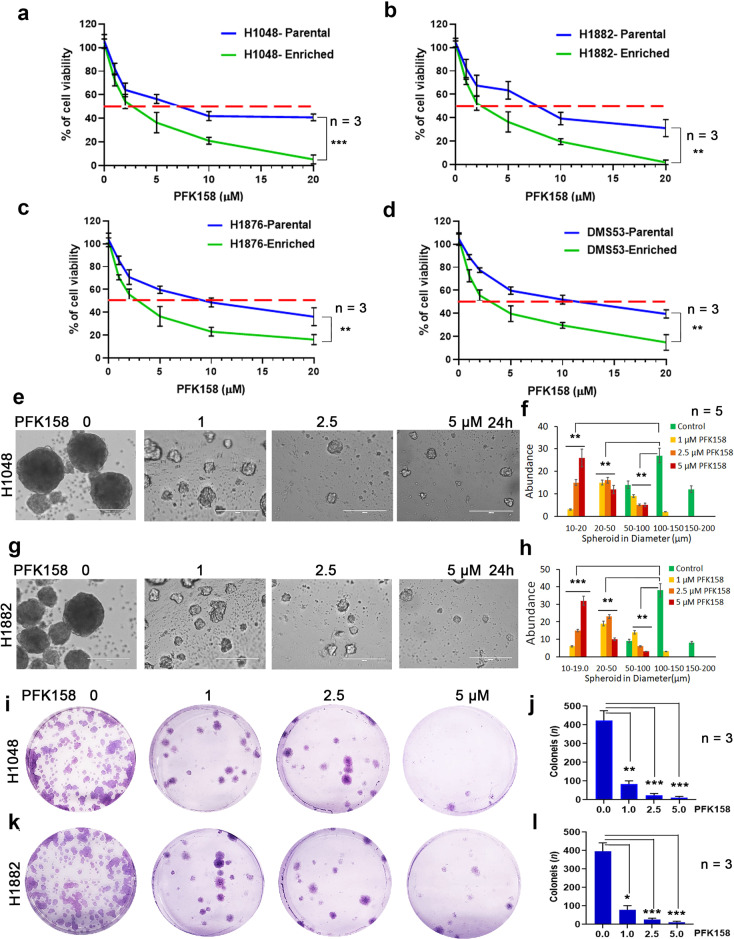
Cytotoxicity of PFK158 on SCLC cells and CSC enriched tumor spheroids. Cell viability was measured following 24 h of PFK158 treatment at 0, 1, 2.5, 5, 10, 20 μM concentrations using MTT assay in both parental and CSC enriched tumor spheroids of (**a**) H1048, (**b**) H1882, (**c**) H1876 and (**d**) DMS53 cells. Values are expressed as the mean ± SD and experiments were conducted in triplicate (*n* = 3) and repeated independently three times. H1048 (**e**) and H1882 (**g**) Cells grown as spheroids were exposed to the indicated concentrations of PFK158 for 24 h and the effects on spheroids were measured (*n* = 5) and (**f** and **h**) Graphs depict the abundance of different size of spheroids upon PFK158 treatment in H1048 and H1882 cells, respectively. Scale bar: 200 μm, ×20 magnification. The effect of PFK158 on colony forming abilities of enriched (**i**) H1048 and (**k**) H1882 cells were assessed and (**j** and **l**) The number of colonies was counted and plotted (*n* = 3) in triplicates. Data are shown as mean ± SD and significance was determined comparing test samples to untreated control and expressed as *p < 0.05, **p < 0.01, ***p < 0.001.

PFKFB3 inhibitor PFK158 has been reported to inhibit the progression of several types of cancer cells at relatively low concentrations [29–31]. To determine if PFKFB3 expression has selective impact on CSC enriched cells, we examined the cell viability effects of PFK158 on SCLC cells and observed a dose-dependent suppression of cell proliferation after 24 h for H1048, H1882, H1876, and DMS53 cells (IC_50_: H1048, 7.1 ± 1.6 μM; H1882, 8.4 ± 2.8 μM; H1876, 10.1 ± 2.3 μM; DMS53, 11.2 ± 2.4 μM). In parallel, these cell lines were grown in medium to enrich CSCs and under 3D culture conditions for a week, which resulted in enhanced sensitivity to PFK158 (IC_50_: H1048, 2.2 ± 0.8 μM; H1882, 2.6 ± 1.2 μM; H1876, 3.06 ± 0.8 μM; DMS53, 3.6 ± 1.1 μM) (Fig. 2a–d). In contrast, this enriched population was less sensitive to 5-FU treatment (Supplementary Fig. 2a–d), which suggests that the CSC-enriched population is more sensitive to PFK158. The effect of PFK158 on the CSC-enriched population was further assessed by 3D spheriod culture and colony formation assays. PFK158 significantly inhibited spheriod formation by both the quantity and the size of spheroids (Fig. 2e–h) and colony forming ability (Fig. 2i–l) of H1048 and H1882 in a concentration-dependant manner.

### PFK158 inhibits glucose uptake and lactate/ATP production in CSC-enriched SCLC cells

**Fig. 3:**
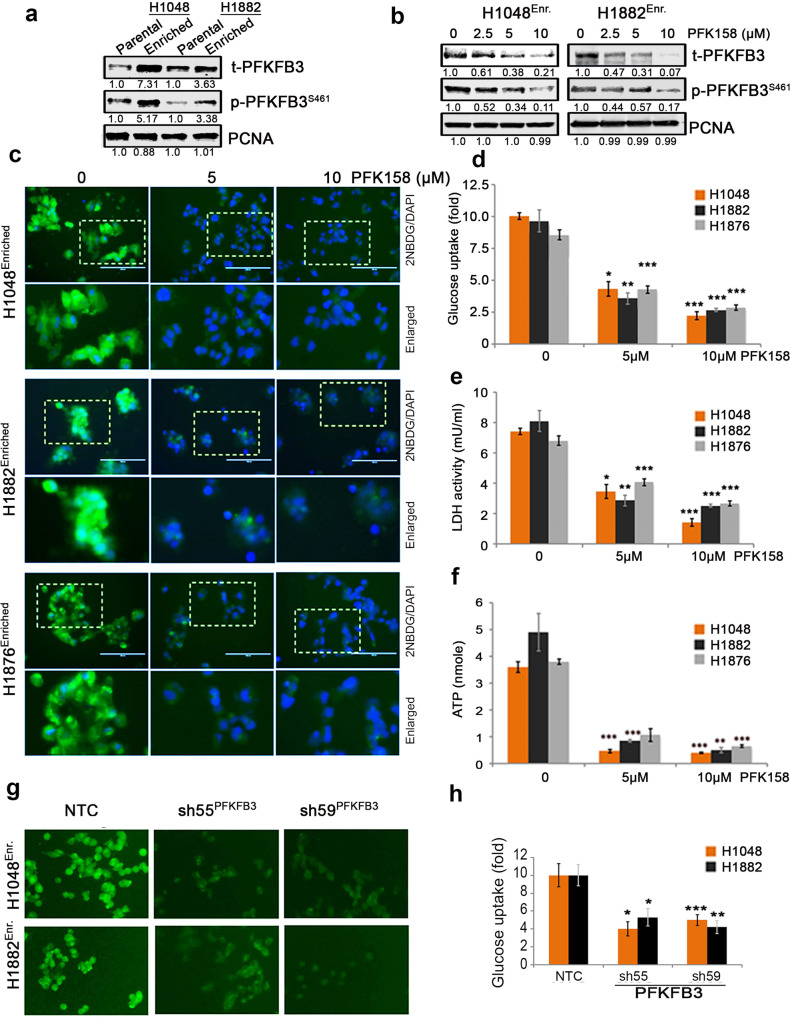
Pharmacological PFKFB3 inhibition with PFK158 suppresses the glycolytic activity of SCLC cells in vitro. **a** Expression levels of total (t)-PFKFB3 and phospho (p)-PFKFB3 (ser461) in parental (epithelial-2D culture) and enriched (spheroidal 3D culture) conditions of H1048 and H1882 cells by immunoblot (IB) analysis. **b** Expression of t-PFKFB3 and p-PFKFB3 (ser461) in H1048 and H1882 3D-spheroids were determined after PFK158 (0, 2.5, 5, 10 μM) treatment for 24 h by IB analysis. Densitometric analysis showing fold change was calculated using Image J software. **c** Glucose uptake was analyzed using 2-NBDG in CSC enriched spheroids of H1048, H1882 and H1876 after treatment with PFK158 at indicated concentrations (Scale bar: 100 μm, ×40 magnification) and (**d**) Pictograph shows percentage of glucose uptake. Intracellular LDH activity (**e**) and ATP generation (**f**) were measured in H1048, H1882 and H1876 cells with or without PFK158. **g** Genetic knockdown of PFKFB3 inhibits glucose uptake in spheroids of H1048 and H1882 (Scale bar, 100 μm) and (**h**) Bar graph shows percentage of glucose uptake. PCNA used as loading control. All experiments were repeated at least three times (*n* = 3). Data are shown as mean ± SD and significance was determined comparing test samples to untreated control and expressed as *p < 0.05, **p < 0.01, ***p < 0.001.

It is well known that increased glucose uptake and aerobic glycolysis is a characteristic feature of CSCs, which regulates thier progression and self-renewal [32]. The level of total PFKFB3 and phospho-PFKFB3 S461 protein was increased in parental cells (2D) and CSC-enriched spheroids (3D) (Fig. 2a). Incremental PFK158 doses decreased total and phospho-PFKFB3 S461 in enriched SCLC cells (Fig. 3b). To assess the effect of PFK158 on glycolysis inhibition in the CSC-enriched population, the uptake of the 2NBDG glucose analog was measured in enriched H1048, H1882, and H1876 cells (Fig. 3c, d). PFK158 treatment also corresponded to a reduction in lactate dehydrogenase activity (Fig. 3e) and intracellular ATP levels (Fig. 3f). These results suggests PFK158 inhibits the glycolytic pathway in enriched SCLC cells. Also, knockdown of PFKFB3 resulted in decreased glucose uptake (Fig. 3g, h), and lactate dehydrogenase activity and intracellular ATP levels (Supplementary Fig. 3a, b) compared to NTC control cells. Collectively, these results confirm the role of PFKFB3 in modulating the glycolytic rate in CSC-enriched SCLC cells.

### PFKFB3 inhibition decreases ABCG2 expression and enhances the CSC-enriched SCLC response to chemotherapeutic drugs

**Fig. 4:**
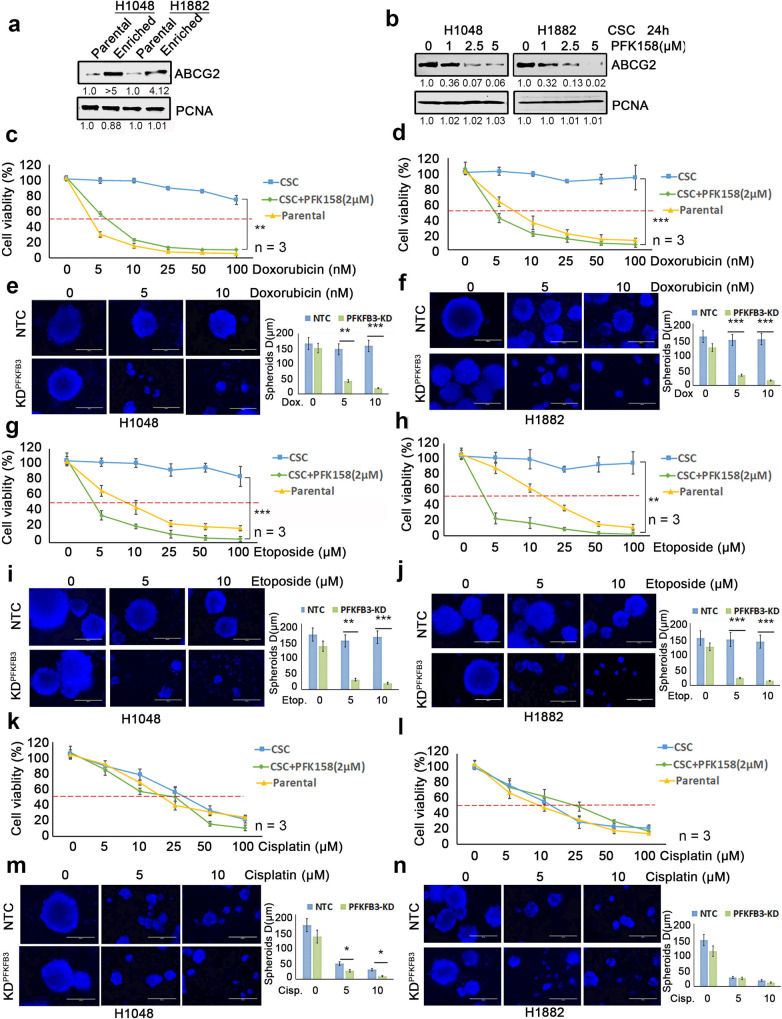
Loss of PFKFB3 induces synergistic activity with ABCG2 substrate chemo therapeutic agents to inhibit CSC mediated proliferation of SCLC cells. **a** The expression levels of ABCG2 in parental (2D culture) and enriched CSC (spheroidal 3D culture) conditions of H1048 and H1882 cells by western blot analysis. **b** Inhibition of ABCG2 in H1048 and H1882 CSC (Aldh1^high^/CD133^high^) cells were determined after PFK158 (0, 1, 2.5, 5 μM) treatment for 24 h by IB analysis. Both 2D (parental) and 3D-hangdrop (CSC) H1048 (**c**) and H1882 (**d**) cells were incubated with Doxorubicin at specified range of concentrations in plus or minus PFK158 (2 µM) for 24 h and cell viability was measured by Trypan blue dye exclusion assay. Depletion of PFKFB3 by shRNA enhances the sensitivity of H1048 (**e**) and H1882 (**f**) CSC cells to Doxorubicin at different concentration and graph shows range of tumor spheres size in NTC versus PFKFB3 knockdown. Both 2D and 3D type H1048 (**g**) and H1882 (**h**) Cells were incubated with for Etoposide (0–100 µM) with or without PFK158 for 24 h and cell viability was measured. Knock down of PFKFB3 sensitize H1048 (**i**) and H1882 (**j**) CSC cells to Etoposide and reduces tumor-sphere sizes (respective bar graphs). Cisplatin (0–100 µM) induced cytotoxicity on Parental and CSC cells of H1048 (**k**) and H1882 (**l**) cells were measured in presence or absence of PFK158 for 24 h. Genetic inhibition of PFKFB3 enhances sensitivity of H1048 (**m**) and H1882 (**n**) CSC cells to Cisplatin with reduced tumor-sphere sizes (respective bar graphs). All experiments were performed in triplicate (*n* = 3) and Data are represented as mean ± S.D. Significance measured comparing individual groups with control is represented as *p < 0.05, **p < 0.01, ***p < 0.001. Magnification ×20, Scale 200 µm.

ABCG2, a member of the ABC transporter family, is reported to efflux a wide variety of endogenous and exogenous compounds from cells while also promoting stem cell proliferation and maintenance of the stem cell phenotype [16, 18]. Western blot analysis showed that ABCG2 is expressed at a very high level in the enriched CSC population compared to parental cells (Fig. 4a). Also, genetic KD and PFK158-mediated inhibition of PFKFB3 downregulates ABCG2 expression (Fig. 4b and Supplementary Fig. 1b). Since PFK158 treatment was found to reduce CSC markers, induce apoptosis, and decrease ABCG2 in the enriched SCLC cells, we tested if combining PFK158 with antineoplastic drugs would result in a synergistic response for CSC cells. PFK158 was combined with doxorubicin, etoposide, or cisplatin in ABCG2^low^ (parental H1048 and H1882) and ABCG2^high^ (H1048^CSC^ and H1882^CSC^) in 2D and 3D-hangdrop viability assays, respectively. PFKFB3 degradation by PFK158 or genetic knockdown potentiated the sensitivity of ABCG2^high^ CSC cells to ABCG2 substrate anticancer drugs in vitro (Fig. 4c–n). While ABCG2-expressing CSC cells were resistant to increasing doses of doxorubicin (Fig. 4c, d), etoposide (Fig. 4g, h), and 5FU (Supplementary Fig 4a, b), PFK158 substantially reduced cell viability by 80% for ABCG2^high^ CSC cells. However, the ABCG2 non-substrate drugs paclitaxel and cisplatin had cytotoxicity responses similar to ABCG2^low^ parental cells (Fig. 4k, l, Supplementary Fig 4c, d). In combination, PFK158 (2 µM) was found to significantly potentiate the anticancer activity of ABCG2 substrate anticancer drugs (doxorubicin, etoposide, and 5FU) in ABCG2^high^ H1048^CSC^ and H1882^CSC^ cells (Fig. 4c, d, g, h) and no notable synergistic combination effect was observed with the non-substrate drugs in ABCG2^high^ CSC cells. Also, we found that knockdown of PFKFB3 sensitizes the CSC cells to the ABCG2-substrate drugs doxorubicin (Fig. 4e, f) and etoposide (Fig. 4i, j) when compared to non-substrate drug cisplatin (Fig. 4m, n). These results suggest that PFK158 can enhance the response of chemotherapy drugs in cells that utilize the ABCG2 transporter to maintain cancer stemness.

### Counteraction of PFKFB3 significantly impairs SCLC pluripotency targeting YAP/TAZ signaling

**Fig. 5:**
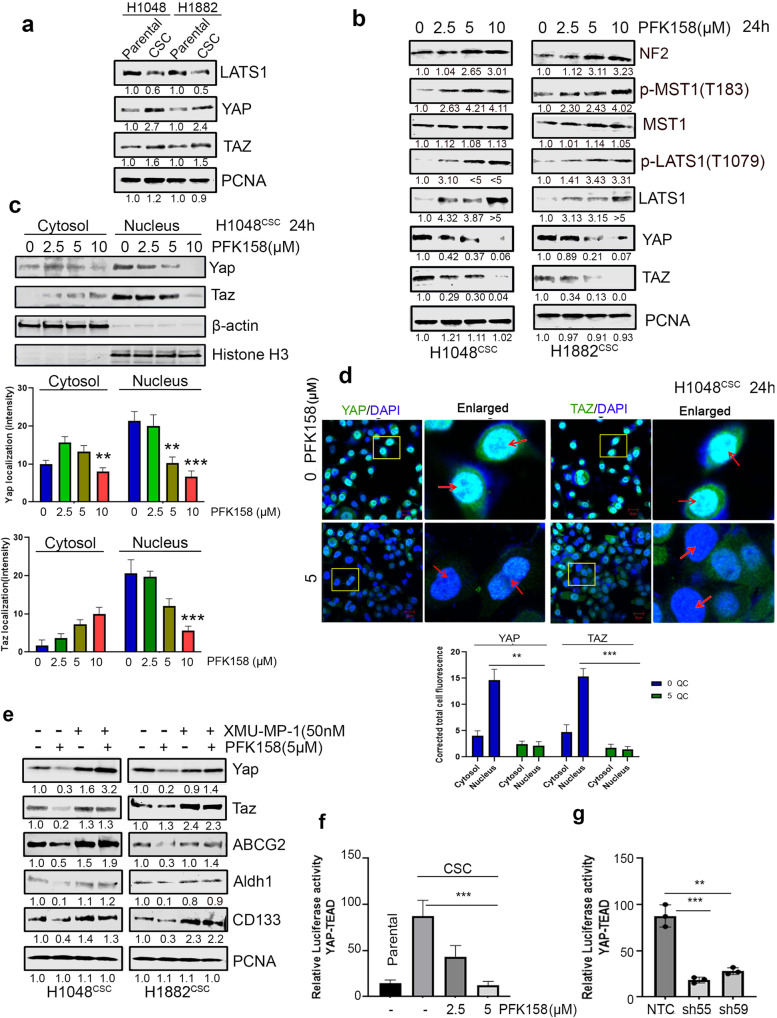
Impairment of PFKFB3 significantly reticences SCLC pluripotency targeting YAP/TAZ expression. **a** Immunoblot analysis of level of LATS1 and YAP/TAZ in parental and CSC (Aldh1^high^/CD133^high^) of H1048 and H1882 cell lines. **b** Inhibition of YAP/TAZ via p-LATS1(T1079) activation through p-MST1(T183) and NF2 by PFK158 at specified concentrations was verified by western blot analysis. **c** Nuclear localization of YAP/TAZ in H1048^CSC^ after exposure to PFK158 was assessed by cytosolic and nuclear fractionation studies and bar graphs shows the level of YAP/TAZ in cytosol vs nucleus. **d** Representative confocal images show PFK158 mediated nuclear translocation of YAP/TAZ in H1048^CSC^ cells and bar graph shows the corrected total cell fluorescence for YAP/TAZ localization. **e** YAP/TAZ activator XMU-MP-1 reverses PFK158 mediated inhibition of ABCG2 and CSC markers Aldh1/CD133 in H1048^CSC^ and H1882^CSC^ cells. **f** H1048^CSC^ co-transfected with pGL3b-YAP/TAZ -TEAD and pNL1.1.TK constructs and then treated with PFK158 (5 µM) for 24 h and activity reported as relative luminescence units (RLU). **g** H1048^CSC^ NTC, PFKFB3 KD cells co-transfected with pGL3b-YAP/TAZ--TEAD and pNL1.1.TK constructs and luciferase activity reported. PCNA, β-actin or histone H3 are used as loading control. Data are represented as mean ± S.D. Significance measured comparing individual groups with control is represented as *p < 0.05, **p < 0.01, ***p < 0.001. Magnification x20, Scale 200 µm.

Pluripotent CSC cells are endowed with higher level of active YAP/TAZ expression compared with differentiated cancer cells [9, 15], and recently Dai et al. reported that upregulation of YAP/TAZ pathway promoted drug resistance through regulating ABCG2 in lung cancer [33]. Our results showed augmented YAP/TAZ expression in CSC compared to SCLC epithelial cells (Fig. 5a). Neurofibromin-2 (NF2) promotes tumor suppression in the LATS1/2 canonical hippo pathway through MST1. Active phospho-LATS1/2 will cause subsequent phosphorylation of YAP1, which leads to YAP1 ubiquitination [34–36]. We further used to immunoblot to uncover whether downregulation of PFKFB3 has a role against ABCG2 expression via regulating YAP/TAZ activation while PFKFB3 was targeted with PFK158 in H1048^CSC^ and H1882^CSC^. Downregulation of PFKFB3 leads to inhibition of YAP/TAZ expression by phosphorylating LATS1 at T1079 through phospho-MST1 (T183) and NF2 overexpression (Fig. 5b), and no remarkable changes in LATS2 level were detected (Supplementary Fig. 5a). Also, we found cytoplasmic retention of YAP/TAZ proteins in H1048^CSC^ (Fig. 5c) by subcellular fractionation blots and confocal imaging, which shows that impaired nuclear accumulation of YAP/TAZ by PFK158 (Fig. 5d, and graphs show the YAP/TAZ localization intensity). Further, to understand the role of YAP/TAZ in PFKFB3 mediated regression of cancer stemness and ABCG2 expression, XMU-MP-1 (an inhibitor that activates YAP/TAZ via MST1/2 inhibition [37]) used to activate YAP/TAZ in H1048^CSC^ and H1882^CSC^ cells and when treated with PFK158. Results highlighted that in the absence of hippo inhibitor XMU-MP-1, PFK158 treatment inhibits the YAP/TAZ, ABCG2, and CSC marker expressions. However, the presence of XMU-MP-1 activated YAP/TAZ reversed the PFK158 mediated counteraction of ABCG2 and CSC markers expression (Fig. 5e).

Furthermore, by performing a dual YAP/TAZ-TEAD luciferase reporter assay, we demonstrated that compared to control or NTC-CSC cells, downregulation of PFKFB3 by shRNA/PFK158 treatment suppressed the YAP/TAZ transcriptional activity as measured by relative luciferase activity (Fig. 5f, g). As ABCG2 is YAP/TAZ transcriptional target gene, enriched SCLC cells were treated with YAP/TAZ-TEAD specific inhibitor MGH-CP1 in presence or absence of ABCG2 substrate drugs doxorubicin and etoposide. However, MHG-CP1 not significantly improved the susceptibility of CSC to chemotherapeutics as it is failed to decrease CSC markers, though ABCG2 level decreased (Supplementary Fig. 5b–d). Collectively, these results suggested the repression of YAP/TAZ by regulating PFKFB3 is critical in inhibiting CSC/therapeutic resistance of SCLCs.

### PFK158 arrests the epithelial-to-mesenchymal transition, migration, and invasion of SCLC cells

**Fig. 6:**
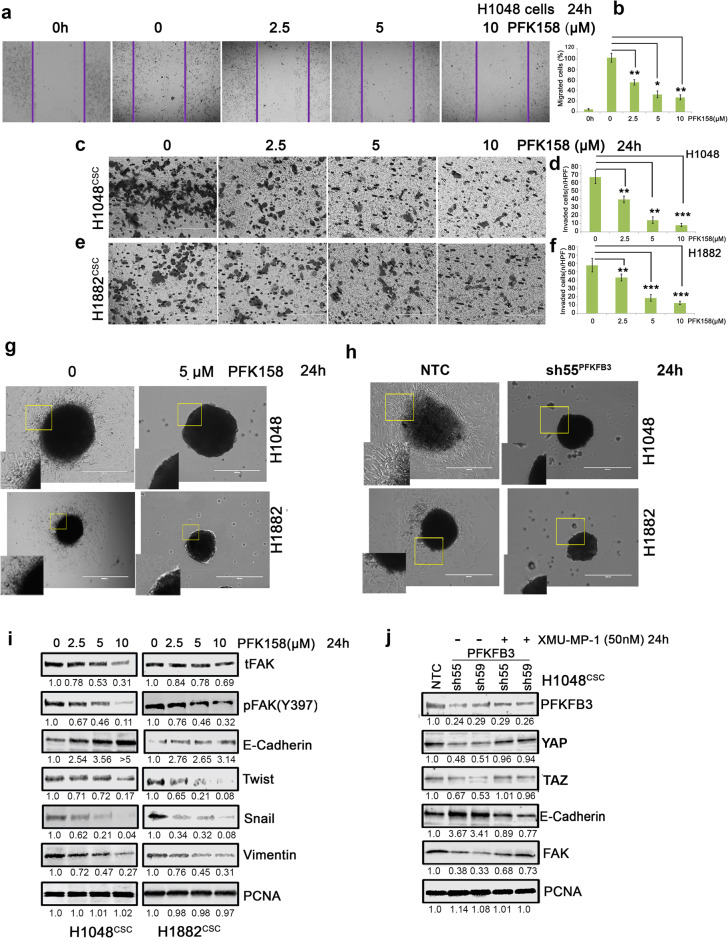
Pharmacological impairment of PFKFB3 inhibits migration/invasion of SCLC. **a** The effect of PFK158 on H1048 motile ability was measured by scratch wound healing assay and (**b**) Pictogram shows PFK158 exhibited migration inhibition (Magnification x20 and Scale bar 200um). Transwell migration assay of (**c**) H1048 and (**e**) H1882 CSC (Aldh1^high^/CD133^high^) cells treated with PFK158 at 0 2.5, 5,10 µM for 24 h (Magnification x40 and Scale bar 100 um). (**d** and **f**) Bar graphs represent the mean account of cells invaded high per field. (**g** and **h**) Validation of tumor spheroid invasion distance migrated by H1048 and H1882 after inhibition of PFKFB3 with PFK158 or knockdown, using 3D tumor spheroid invasion assay. **i** Concentration dependant inhibition of epithelial to mesenchymal transition (EMT) marker expressions upon PFK158 treatment in H1048 and H1882 3D-spheroids were verified by immunoblot analysis. **j** YAP/TAZ activator XMU-MP-1 reverses PFKFB3^KD^ mediated regression of EMT markers in H1048^CSC^ cells. PCNA was used as loading control. Statistical significance calculated by comparing test group with untreated control and expressed as *p < 0.05, **p < 0.01, ***p < 0.001.

EMT is a key regulator of cell invasion and metastasis in cancers. In addition to the acquisition of migratory/invasive abilities, the EMT process is tightly connected with the generation of CSCs and development of chemoresistance [14, 15, 32]. Following promising inhibition of cancer stemness and ABCG2 levels, an impact of PFKFB3 depletion by PFK158 or its genetic KD effect on migration and invasion was examined on H1048 and H1882 cells through invasion and migration assay and EMT protein expression analysis by immunoblot. Results conferred that PFK158 decreased the migration rate in a dose-dependent manner (47.6%, 71.2%, & 77.8%, Fig. 6a, b) in H1048 cells. Similar attenuation of the invasion phenomenon was found with 39 ± 5 to 9 ± 3 cells/HPF in H1048^CSC^ when untreated showed 66 ± 8 cells/HPF (Fig. 6c, d) and with 43 ± 6 to 13 ± 4 cells/HPF in H1882 when compared to that of untreated (57 ± 8 cells/HPF) (Fig. 6e, f). To further validate the role of PFKFB3 in preventing invasion of the enriched SCLC cells, we performed the spheroid invasion assay. PFK158 treatment or PFKFB3 knockdown potentially impaired the sprouting of the invading cells from the spheroids compared to control or non-targeted cells respectively (Fig. 6g, h). Immunoblot analysis showed that PFK158 treatment reduced the expression levels of critical EMT markers including Twist, Snail, and vimentin along with gradual upregulation of E-cadherin expression and impaired levels of phospho-FAK Y397 and total FAK expression (Fig. 6h). Furthermore, to understand the role of YAP/TAZ in regulating EMT markers, PFKFB3-KD H1048^CSC^ and H1882^CSC^ cells were treated with XMU-MP-1. Results showed that treatment of XMU-MP-1 reversed the PFKFB3 depletion associated EMT markers reduction by rescuing the YAP/TAZ pathway activation (Fig. 6i). Together, these results suggested that the repression of PFKFB3 has an imperative role in migration/invasion of SCLCs by negatively regulating YAP/TAZ and EMT marker levels.

### PFKFB3 inhibition and knockdown inhibits tumor growth in a SCLC xenograft model

**Fig. 7:**
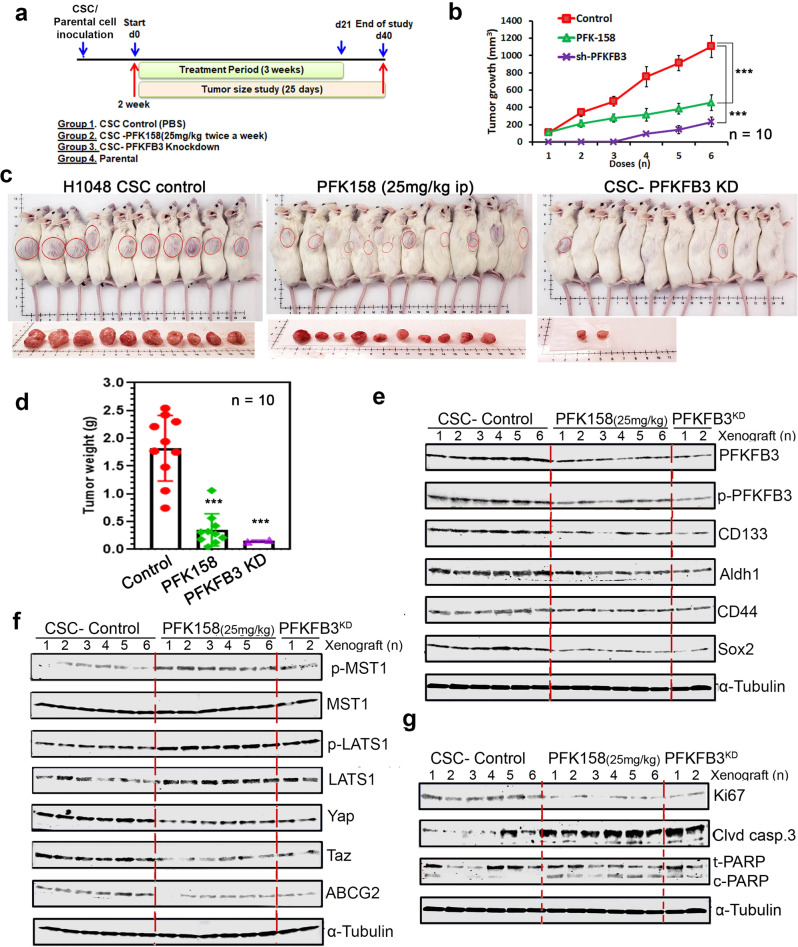
Antitumor role of PFKFB3 in PFK158 treatment or PFKFB3 knock down in SCLC xenograft model. **a** Schematic diagram displaying the time course of tumor induction and treatment in mice. **b** Representation of tumor growth inhibition by PFK158 and PFKFB3 genetic down regulation **c** Physical morphology and tumor images of xenograft from each group are shown. **d** Effect of pharmacological and genetic down regulation of PFKFB3 on tumor growth in NSG mice (*n* = 10 per group) and final tumor weights from different groups are shown. **e** Western analysis of the effect of PFKFB3 deficiency on cancer stemness markers including Aldh1, CD133, CD44, and Sox2. **f** Western blot analysis was performed to assess phospho and total LATS1/MST1, YAP/TAZ, and ABCG2 expression in lysates from the randomly selected xenografts in each group. **g** Immunoblot analysis of proliferative and apoptotic markers expressions such as Ki67, cleaved caspase3 and PARP from tumors of each group. α-tubulin was used as loading control. Each bar represents the mean ± SD (*n* = 10), Significant value expressed as *p < 0.05, **p < 0.01, and ***p < 0.001 by comparing test groups with control group.

The pathophysiological response of PFK158 was investigated to determine whether PFKFB3 perturbation affects CSCs induced tumor growth in vivo. A SCLC xenograft model was established by subcutaneously injecting H1048^Parental^, H1048^CSC^ and H1048^shPFKFB3-CSC^ cells. After 2 weeks, PKF158 (25 mg/kg, twice/week for 6 doses, *i.p*.) was administered and tumor growth was recorded. Mice injected with H1048 parental cells did not develop tumors until the end of the study (Fig. 7a). The CSC generated tumors with PFK158 treatment or knockdown of PFKFB3 significantly reduced tumor establishment as measured by vernier caliper and gradually inhibited tumor growth in dose dependent manner (Fig. 7b). Anatomical appearance of flank-containing tumor shows that inhibition of PFKFB3 by PFK158 and genetic knockdown with shPFKFB3 regressed CSC tumor size noticeably with 0.353 ± 0.28 g and 0.135 ± 0.025 g of tumor respectively, when corresponded to that of untreated (1.80 ± 0.6 g) (Fig. 7c, d). Both the administration of PFK158 or genetic knockdown of PFKFB3 significantly prevents the tumor formation of SCLC cells in vivo.

### PFKFB3 inhibition impairs SCLC pluripotency by diminishing activation of YAP/TAZ pathway in vivo

**Fig. 8:**
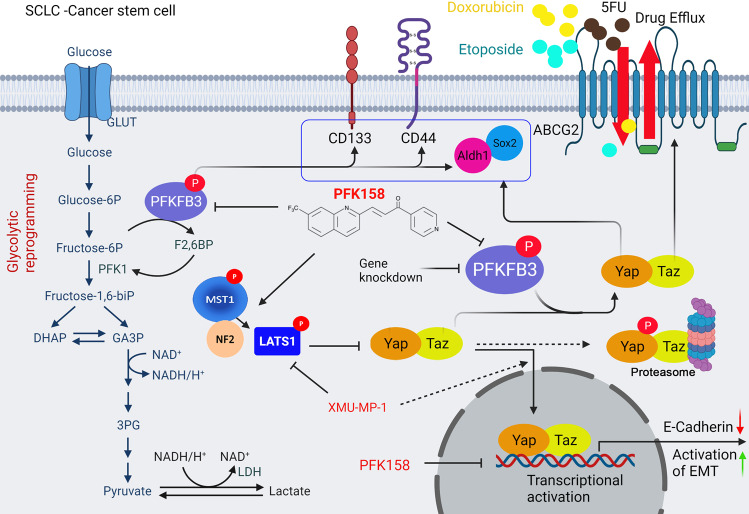
A working model that PFKFB3 inhibition plays an important role of deregulating cancer stemness, EMT and chemosensitivity in small cell lung cancer. PFKFB3 inhibition led to glycolytic rewiring (impaired glycolysis), inhibited the cancer stemness and EMT by downregulating Aldh1, CD133, CD44 and Sox2, and ABCG2 through inactivation of YAP/TAZ pathway which consequently enhanced chemosensitivity and cell death.

The molecular events of PFK158 or PFKFB3 knockdown exhibiting anti-pluripotency in CSC tumor were elucidated by performing western blot in randomly selected 6 tumors from each group. Results demonstrated that inhibition of PFKFB3 with PFK158 or genetic knockdown repressed the expression of phospho-PFKFB3 S461 and total PFKFB3 with decreased in expression of the cancer stem cell markers CD133, CD44, Aldh1, and Sox2 and also reduced expression of YAP/TAZ via upregulation of phospho/total LATS1 and MST1 (Fig. 7e). Also, qPCR results shows that PFK158 treatment decreased the mRNA level of YAP/TAZ target genes including ABCG2, CTGF, SOX2 and Snail (Supplementary Fig. 6a). Our in vivo results corroborated with our in vitro findings as PFK158 treatment or PFKFB3 KD potentially inhibited level of ABCG2 (Fig. 7f) and proliferative marker Ki67 (Fig. 7g) along with an increase in cleaved caspase-3 and cleaved PARP levels indicating induced apoptosis (Fig. 7g). The in vivo effect of PFK158 on EMT markers evaluated by western blot (Supplementary Fig. 6b) showed that PFKFB3 impairment resulted in decrease of vimentin and twist with significant upregulation of E-cadherin, which paralleled the in vitro results (Fig. 6). Taken together, both in vitro and in vivo experimental results summarize that glycolytic reprogramming of impaired PFKFB3 by PFK158 treatment or genetic KD inhibits cancer stemness, migration/invasion, and resistance via regulating critical factors of CSC, EMT, and multidrug resistance, which then led to abolished tumor establishment (Fig. 8).

## Discussion

PFKFB3 expression is reported to be significantly higher in many cancers. In addition to promoting glycolytic function in cancer cells, PFKFB3 was found to be involved in regulating several cellular events, like cell cycle pathway, DNA damage/repair, autophagy, macropinocytosis, and metastasis [25–27, 29, 31]. High PFKFB3 expression was associated with increased tumorigenesis and poor prognosis [38]. Since PFKFB3 was previously reported as an important metabolic target in cancer, we focused our current study to unravel its role in cancer stemness, EMT alteration, and therapeutic resistance by effluxing drugs. Our prior work has shown that inhibition of PFKFB3 with the glycolytic inhibitor PFK158 attenuates tumor progression and increases the efficacy of conventional chemotherapy [29–31]. Herein, we found that PFK158 treatment lead to decreased cell viability in the CSC-enriched population of SCLC cells compared to their parental counterparts (Fig. 2), which suggests that PFK158 selectively targets tumor initiating stem cells.

Cancer cells exhibit increased glucose uptake and the enhanced glycolysis, known as the” Warburg effect,” not only provides the energy for cell survival but also play important role in developing drug resistance [27, 38]. Recent reports showed that CSCs exhibit a unique metabolic phenotype and the glycolytic rewiring in CSCs plays a critical role in stemness and drug resistant phenotype. Therefore, targeting the metabolic switch was proposed to be a new strategy for CSC treatment [27, 39]. Our results show that PFKFB3-mediated targeting by PFK158 or genetic manipulation resulted in inhibition of glycolysis (Fig. 3a–h) and of tumor progression (Fig. 7a–d), resulting in diminished cancer “stemness” by decreasing both surface and intracellular stem cell marker expressions such as CD133, CD44, Aldh1, and Sox2 (Fig. 1a–f). In addition to inhibition of pluripotency, genetic and pharmacological downregulation of PFKFB3 also had an apoptotic-inducing effect on SCLC stem cells (Fig. 1g–i).

Undifferentiated CSCs are endowed with upregulated level of active YAP/TAZ expression compared with differentiated cancer cells [11, 40]. In tumors, increased YAP/TAZ expression or activation disrupts the cell-cell junctions, increases mesenchymal gene expression, and augments the morphological changes associated with EMT. Conversely, loss of YAP or TAZ can arrest EMT [10]. In this study, we found that knockdown of PFKFB3 or inhibition by PFK158 was able to attenuate SCLC-CSC tumor cell migration/invasion (Fig. 6a–g). NF2 is a critical regulator for hippo signaling as its inactivation or ubiquitination impairs LATS1/2 stabilization and activation. NF2 associates with MST1 to facilitate LATS1/2 activation for YAP ubiquitination [34–36]*.* We found that PFKFB3 inhibition by PFK158 induces NF2, which leads to stabilization and activation of total and phospho-LATS1 via MST1, and these results correspond to the YAP/TAZ decrease in protein levels, impaired nuclear localization, and co-transcriptional activity (Fig. 5a–g). However, NF2 can be regulated by many factors and the direct mechanism for upregulation by PFKFB3 remains unknown*.* Impaired YAP/TAZ nuclear entry led to inhibition of EMT markers expressions including Twist, Snail, and vimentin along with phosphorylated FAK and augmented E-cadherin protein levels (Fig. 6h, i).

Numerous molecular mechanisms have been theorized to address the role in drug resistance, including ineffective apoptotic machinery, improved DNA repair signaling, up-regulation of multidrug resistance (MDR) proteins and membrane efflux transporters [5, 16, 18, 41, 42]. Such mechanisms may be mostly responsible for chemoresistant properties of the CSCs and consequent associated recurrence in patients. ABCG2 the efflux pump determines the drug efflux capacity of the CSC sub-population phenotype and is associated with tumor progression, metastasis, and resistance [42–44]. ABCG2 actively effluxes a wide variety of chemically and structurally unrelated compounds from cells including doxorubicin, etoposide, and 5-FU; ABCG2 overexpression in tumor cells confers multidrug resistance to a multitude of anti-cancer drugs levels [17, 43] and a lot of work has been going on to understand and successfully target the ABCG2 induced MDR. Our current study determines that PFK158-mediated inhibition or PFKFB3 KD potentially decreased the level of ABCG2 via down regulating YAP/TAZ by activation of LATS1/MST1 (Fig. 7f) which consequently increased the sensitivity to substrate-anticancer drugs including doxorubicin, etoposide, and 5-FU (Figs. 4 and 7f) whereas no notable synergistic effect was observed with the non-substrate drugs such as cisplatin and paclitaxel (Fig. 4). This result sheds new lights to reverse the resistance and eliminate the CSCs using the ABCG2 substrate-chemotherapeutic drugs with PFKFB3 inhibitors as a combination therapy in SCLC. As YAP/TAZ inhibition is associated with ABCG2 expression, YAP/TAZ specific inhibitor MGH-CP1 were treated with ABCG2 substrate drugs doxorubicin and etoposide. However, MHG-CP1 not significantly enhanced the sensitivity of CSC to chemotherapeutics as it is failed to decrease CSC markers, though ABCG2 level decreased (Supplementary Fig. 5b–d). Therefore, repression of YAP/TAZ by regulating PFKFB3 may be advantageous for overcoming CSC/therapeutic resistance in SCLC.

In addition, a SCLC xenograft model was used to evaluate the role of PFKFB3 in CSC mediated tumor growth in vivo. Inhibition with PFK158 or genetic knockdown of PFKFB3 showed reduced tumor formation in the CSC xenograft model with reduced expression of phospho-PFKFB3 S461 and total PFKFB3, and cancer stem cell markers including CD133, CD44, Aldh1, Sox2, and ABCG2 (Fig. 7a–f). PFKFB3 impairment also showed attenuated expression of YAP/TAZ via upregulation of pLATS1 through pMST1/NF2, which led to decrease of vimentin and twist with significant upregulation of E-cadherin (Fig. S5b), which is corroborated to in vitro results (Fig. 6a–i).

In conclusion, we confirmed that either genetic downregulation or pharmacological inhibition of PFKFB3 exhibited anti-glycolytic effect in SCLC CSC cells and further demonstrated that the antitumor activities is associated with loss of pluripotency and drug efflux, impaired cell invasion and apoptosis in SCLC tumor models (Fig. 8). Since cancer stemness is a primary mechanism for resistance to treatment and promoting metastasis, targeting CSC metabolism can be a selective approach against tumor recurrence in patients.

## Materials and methods

### Reagents and antibodies

Reagents and antibodies are listed in Supplementary Table 1.

### Cell culture

Human small cell lung carcinoma (SCLC) cell lines NCI-H1048, NCI-H1882, NCI-H1876, DMS53 from American Type Culture Collection (ATCC) (VA, USA) were grown in DMEM/F12 media (Gibco) supplemented with 5% fetal bovine serum (R&D Systems), 10 nM Hydrocortisone, 10 nM β-estradiol, 1x-ITS (Insulin-Transferrin-Sodium selenite), 2 mM L-glutamine and 1% penicillin-streptomycin 5% CO_2_ humidified atmosphere at 37 °C and treated with PFK158 at different concentrations (0–20 µM) for 24 h and cytotoxicity was measured by MTT assay and clonogenic assay as reported earlier [45].

### Hanging drop 3D method

SCLC cells were cultured and seeded at 2000 ± 20 cells per 30 µL drop of cancer stem cell media (Promo Cell, #28070) on the inner side of a 10 cm dish lid. The lid was downturned, placed on top of a plate filled with PBS for 7 days to spot the spheroid [46] Each spheroid was treated with PFK158 at (0-20 µM) in triplicates for 24 h and cell viability was assessed by trypan blue dye exclusion method [47].

### Generation of PFKFB3 knockdown cells

PFKFB3 downregulation was performed in H1048 and H1882 cells with shPFKFB3 [Sh55: CGGGTGCATGATTGTGCTTAA (targeting 3′UTR), Sh59: CCACCAATACTACTAGAGAGA (targeting 5′UTR)] using Lipofectamine 3000 (Invitrogen) as per manufacturer protocol and non-targeted clones (NTC) and knockdown (KD) clones were generated as reported earlier [48] for further studies.

### Spheroid formation assay and drug combinations

H1048 and H1882 or corresponding NTC, sh55^PFKFB3^, sh59^PFKFB3^ cells were cultured, trypsinized, and resuspended in cancer stem cell media or DMEM/F12 supplemented with 20 ng/mL EGF, 10 ng/mL bEGF, and B27 and 5 × 10^4^ cells / well were seeded into 6-well ultra-low adhesion plates (Corning, New York, USA). The cells were cultured for a week for enrichment of CSC characteristics and exposed with or without PFK158 (Gossamer Bio, San Diego, California), or in combination with doxorubicin, etoposide, 5-FU, cisplatin and paclitaxel, then spheres with diameter 10-200μm in different fields were counted and stained with DAPI and imaged. YAP/TAZ inhibitor MGH-CP1 also tested in combination with chemotherapeutics against CSC proliferation. For western blot analysis, lysates were prepared from spheroids treated with or without PFK158 in plus or minus XMU-MP-1 at various conditions by using cell lysis buffer (Cell Signaling #9803S).

### Glucose uptake assay

H1048 and H1882 or corresponding NTC, sh55^PFKFB3^, sh59^PFKFB3^ cells were cultured as spheroids and treated with PFK158 (0, 5, 10 µM) for 24 h, followed by the incubation with 2-NBDG (150 µg/mL) for 30 min in the glucose-free medium and imaged with fluorescent intensities calculated using ImageJ software [31].

### Lactate dehydrogenase (LDH) activity assay

H1048 and H1882 cells were cultured as spheroids in the absence and presence of PFK158 to induce cytotoxicity and subsequently release LDH. LDH activity was measured according to the manufacturer’s protocol (BioVision Inc., Milpitas-CA, USA).

### Measurement of ATP production

H1048 and H1882 (1 × 10^6^) cultured in ultra-low attachment 6-well plate for enrichment and treated with PFK158 at 0, 2.5, 5 μM for 24 h and ATP production was measured by using ATP assay kit following the manufacturer’s protocol (BioVision Inc., Milpitas CA-USA).

### Detection of CSC surface markers by FACS

The CSC enriched H1048 and H1882 cells untreated or were treated with variable doses of PFK158 for 24 h, washed once with ice-cold sterile PBS and were labeled with PE-CD133 for 30 min at 4 °C in the dark. Cells were washed twice with 1% FBS containing PBS and finally resuspended in the assay buffer for surface marker detection on the BD FACSAria II Flow Cytometer and analyzed in FlowJo software.

### Aldefluor (Aldh1) assay

To identify the cell population with Aldh1 enzymatic activity, we used the Aldefluor kit (Stem Cell Technologies, Vancouver, Canada) following manufacturer’s instructions. In brief, CSC enriched SCLC cells were treated with PFK158 (0–10 µM) for 24 h and 1 × 10^6^ cells were resuspended in 1 mL Aldefluor assay buffer. Following the addition of 5 µl of the activated Aldefluor reagent to control and test sample, then cells were incubated for 30 minutes at 37 °C, washed and resuspended in 500 µL Aldefluor assay buffer were sorted on the BD FACSAria II Flow Cytometer and analyzed in FlowJo.

### Annexin V-Pacific blue/PI dual staining

The Annexin V-Pacific blue/PI dual staining assay (Life Technologies) was employed to assess the PFK-158 mediated cell death according to protocol provided by the manufacturer. In brief, approximately 1 × 10^6^ H1048^CSC^ and H1882^CSC^ cells were grown as CSC enriched spheroids and treated with PFK158 (0, 2, 5 µM) for 24 h. The cells were sorted using a flow cytometer (BD FACS Canto II) and analyzed using FlowJo [49].

### Matrigel transwell and spheroid invasion assays

Transwell invasion assay was employed to assess the anti-invasive role of PFKFB3 inhibition on H1048^CSC^ and H1882^CSC^ cells as reported before [45]. In brief, serum-free medium containing 5 ×10^4^ of H1048^CSC^ and H1882^CSC^ cells with PFK158 (0, 3, 5, 10 µM) cultured onto top of invasion chamber coated with ECM gel and complete medium (10% FBS) was added in the lower chamber, incubated for 24 h. Then lower chambers were fixed with methanol, stained with crystal violet (0.4 g/L) and invaded cells were counted in Olympus Phase Contrast microscope. Tumor spheroids of H1048 and H1882 or PFKFB3 KD cells were sandwiched in matrigel as reported earlier [50] and complete medium containing plus or minus PFK158 added on the top of matrigel, then incubated for 24 h and invading cells were visualized in inverted microscope.

### Migration assay

Anti-migration effect of PFK158 on H1048 cells was assessed by scratch wound healing assay as reported earlier [51]. A monolayer of H1048 cells was scratched to form a wound and exposed with PFK158 (0, 2.5, 5, 10 µM), incubated for 24 h, fixed with methanol and stained in crystal violet Cell migrations were photographed at identical locations. Percentage of cell migration was calculated by comparing final gap width to initial gap width

### DLR assay

To determine the effect of YAP/TEAD promoter activity, enriched H1048 cells were cotransfected with reporter constructs such as 8xGITTC-luciferase (pGL3b-Yap/Taz) (Addgene, plasmid #34615, Cambridge, MA, USA) and the pNL1.1.TK vector and after 24 hours post transfection, luciferase activity was measured with dual luciferase reporter assay (Promega#1960) according to the manufacturer’s recommendations. Relative firefly luciferase activity was normalized to renilla luciferase activity.

### Quantitative real-time PCR

Quantitative real-time PCR was performed using iQTM SYBR Supermix (Bio-Rad, Hercules, CA, USA) and primers ABCG2 FP: 5ʹ-CTCTTCTTCCTGACGACCAAC-3ʹ ABCG2 RP: 5ʹ-TCCAAGGAAATAAGATGACACTCTG-3ʹ CTGF FP: 5ʹ-CCAATGACAACGCCTCC-3ʹ, CTGF RP: 5ʹ-TTGGAGATTTTGGGAGTACGG-3ʹ, SNAI FP: 5ʹ-ACAAGCACCAAGAGTCCG-3ʹ, SNAI RP: 5ʹ-ATGGCAGTGAGAAGGATGTG-3ʹ, SOX2 FP: 5ʹ-CACACTGCCCCTCTCAC-3ʹ, SOX2 RP: 5ʹ-TCCATGCTGTTTCTTACTCTCC-3ʹ, GAPDH FP:5ʹ-ACATCGCTCAGACACCATG-3ʹ and GAPDH RP:5ʹ-TGTAGTTGAGGTCAATGAAGGG-3ʹ.

### Tumor xenograft study

The animal experiment was performed fulfilling with the guidelines of the Institutional Animal Care and Use Committee at the Mayo Foundation (Protocol #A00003619-18). H1048 and PFKFB3 KD (sh55^PFKFB3^) cells enriched as tumor initiating cells (TIC) for 7 days as described in spheroid culture. Unenriched H1048^Parental^ (10,000 cells/mouse, group-1, *n* = 10), enriched 3D H1048^CSC^ (2500 cells/mouse, group-2 and 3, *n* = 10 each), enriched H1048-sh55^PFKFB3-CSC^ (2500 cells/mouse, group-4, *n* = 10) cells in 200 μL of ECM gel/media (1:1) were injected subcutaneously (sc) on the right flank of the forelimb of SCID-NSG mice. After 100 mm^3^ size of tumor development, mice were randomly assigned to four treatment groups and administered with 40% Captisol (vehicle) for parental group (grp-1), vehicle for control group (grp-2), 25 mg/kg of PFK158 twice in a week for 3 weeks (grp-3) and vehicle for PFKFB3 KD group (grp-4), intraperitoneally (*ip*). After 3 weeks of treatment, all mice were sacrificed, and body weight and tumor volume were measured, and tumors were preserved either in formalin or frozen at −80 °C.

### Western blot analysis

Western blot analysis was performed as described previously [48]. Western blot analysis was performed using cell lysates from SCLC cell lines and xenografts and using antibodies listed in Supplementary Table l and detected by an Odyssey Fc Imaging system (LiCor, USA) and normalized relative expression folds were calculated using Image-J [49].

### Confocal imaging

The enriched H1048 and H1882 cells were grown on multi-chambered slides overnight in cancer stem cell media and then treated with PFK158 at 0 and 5 µM for 24 h. Slides were washed and fixed with methanol, then blocked with 1% BSA in PBS, incubated for 1 h with anti-YAP/TAZ at RT. Primary antibody was detected using rabbit anti-mouse IgG-FITC (Molecular Probes, Eugene, OR, USA) in Zeiss-LSM 510 microscope.

### Statistical analysis

All statistical analyses were performed using GraphPad Prism software (San Diego, CA). Data were analyzed by *t* test. Values were expressed using mean ± standard deviation (SD). Significance was expressed as *p < 0.05 **p < 0.01 ***p < 0.001.

## Supplementary information

Supplementary material
