# Correction to: PFKFB3 regulates cancer stemness through the hippo pathway in small cell lung carcinoma

**DOI:** 10.1038/s41388-022-02470-z

**Published:** 2022-11-28

**Authors:** Prabhu Thirusangu, Upasana Ray, Sayantani Sarkar Bhattacharya, Derek B. Oien, Ling Jin, Julie Staub, Nagarajan Kannan, Julian R. Molina, Viji Shridhar

**Affiliations:** 1 Department of Experimental Pathology and Medicine, Mayo Clinic, Rochester, MN USA; 2 Division of Experimental Pathology, Department of Laboratory Medicine and Pathology, Center for Regenerative Medicine, Mayo Clinic, Rochester, MN USA; 3 Department of Medical Oncology, Mayo Clinic, Rochester, MN USA; 4 Present Address: Oncology R&D, AstraZeneca, Boston, MA USA

**Keywords:** Cell signalling, Cancer stem cells

Correction to: *Oncogene* 10.1038/s41388-022-02391-x, published online 08 July 2022

Following the publication of this article the authors noted a misplaced blot in Figure 1b (for CD133 of H1048 cell line) which is the same figure seen in Figure 1a for Aldh1. A similar misplacement was found in figure 4a (for ABCG2 level) which was repeated in Figure 5e - last two lanes in H1048 (labelled for Yap). The correct version of Figures 1 and 4 are included below.
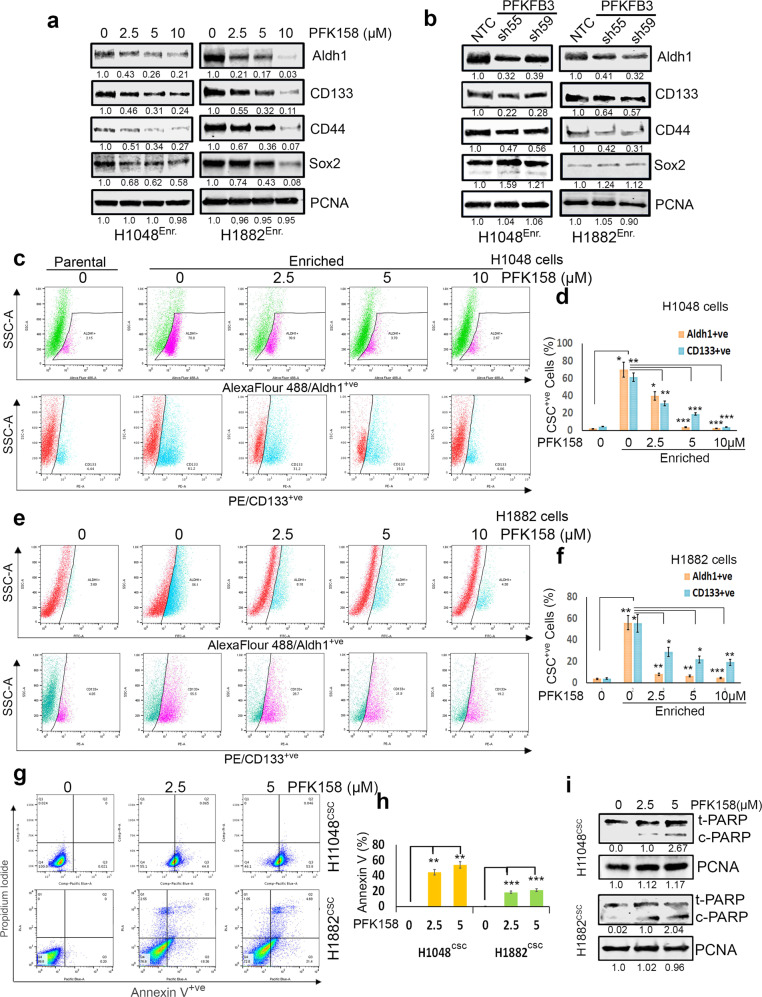

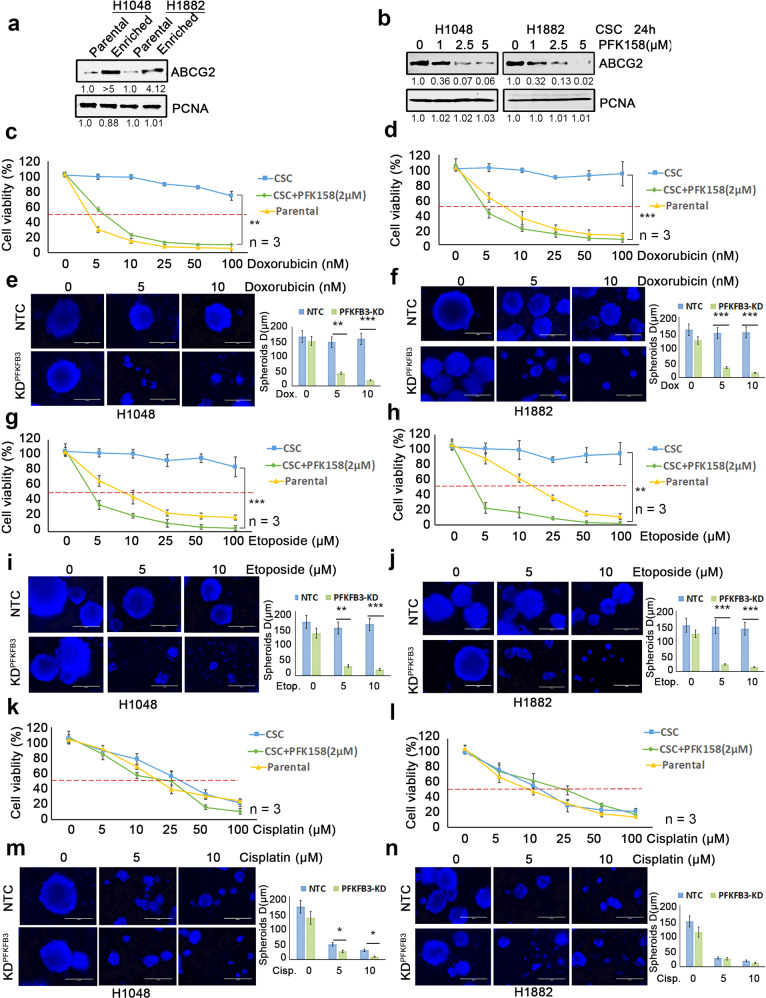


This amendment has no impact on the conclusions of the article. The authors apologise for any inconvenience caused.

The original article has been corrected.

