# Supplementary material for: PFKFB3 regulates cancer stemness through the hippo pathway in small cell lung carcinoma
Source: Oncogene. 2022 Jul 8;41(33):4003–17. doi: 10.1038/s41388-022-02391-x (PMC9374593; doi:10.1038/s41388-022-02391-x)
Supplement: Supplementary file 1 — Supplementary material [file 41388_2022_2391_MOESM1_ESM.docx]

**PFKFB3 regulates cancer stemness through the hippo pathway in small cell lung carcinoma**

Prabhu Thirusangu^1^, Upasana Ray^1^, Sayantani Sarkar Bhattacharya^1^, Derek B. Oien^1†^, Ling Jin^1^, Julie Staub^1^, Nagarajan Kannan^2^, Julian R. Molina^3^ and Viji Shridhar^1^*

**^1^**Department of Experimental Pathology and Medicine, Mayo Clinic, Rochester, MN, USA ^2^Division of Experimental Pathology, Department of Laboratory Medicine and Pathology, Center for Regenerative Medicine, Mayo Clinic, Rochester, MN, USA

**^3^**Department of Medical Oncology, Mayo Clinic, Rochester, MN, USA

^*^Corresponding author: Viji Shridhar ([shridhar.vijayalakshmi@mayo.edu](mailto:shridhar.vijayalakshmi@mayo.edu))

^†^Current address: Oncology R&D, AstraZeneca, Boston, MA, USA

| **Supplementary table.1 List of materials and antibodies used for the study** | | |
| --- | --- | --- |
| **Materials and antibodies** | **Catalog No.** | **Manufacturer/Company** |
| CD133 | #5860S | Cell Signaling Technology, Danvers, MA |
| Sox2 | #23064S |  |
| Vimentin | #5741 |  |
| p-FAK (Y^397^) | #8556 |  |
| t-FAK | #3285 |  |
| E-Cadherin | #3195 |  |
| PARP1 | #9542 |  |
| Cleaved caspase3 | #9661S |  |
| p-MST1(Thr183)/MST2 (Thr180) | #49332S |  |
| MST1 | 14946S |  |
| p-LATS1 (Thr1079) | #8654S |  |
| CD 44 | GTX10211 | GeneTex, CA 92606 USA |
| CD133 PE | 130-110-962 | Miltenyi Biotec, Rhine-Westphalia, Germany |
| t-PFKFB3 | ab181861 | Abcam, Cambridge, UK |
| LATS2 | Ab110780 |  |
| p-PFKFB3 (S^461^) | Custom made | Genescript Inc, NJ, USA |
| ABCG2 | sc-377176 | Santa Cruz Biotechnology, Texas, U.S.A |
| Histone 3 | sc-517576 |  |
| LATS1 | sc-398560 |  |
| Yap | sc-101199 |  |
| Taz | sc-518026 |  |
| Twist | sc-15393 |  |
| α -Tubulin | sc-134237 |  |
| Aldh1 | sc374149 |  |
| NF2 | sc-55575 |  |
| PCNA | sc9857 |  |
| β-actin | sc-517582 |  |
| 2-NBDG | #186689-07-6 | Cayman Chemicals, Ann Arbor, MI |
| 3-(4,5-dimethylthiazol-2-yl)-2,5-diphenyltetrazolium bromide (MTT) | M6494 | ThermoFisher Scientific, Waltham, MA, USA |
| Propidium iodide | P1304MP |  |
| pLATS2 (Thr1041) | PA5-117227 |  |
| ATP Colorimetric/Fluorometric Assay Kit | #K354 | Biovision Inc, Milpitas, CA |
| LDH activity colorimetric assay kit | # K726-500 |  |
| Doxorubicin | D1515 | Sigma Aldrich, Burlington,  USA |
| Paclitaxel | T7191 |  |
| 5-Flourouracil | F6627 |  |
| Dual-Luciferase® Reporter Assay System | E1960 | Promega, Madison, WI, USA |
| MGH-CP1 | S9735 | Selleckchem, Houston, TX, USA |
| XMU-MP-1 | HY-100526 | MedChemExpress, NJ 08852, USA |
| Etoposide | #341205 | Calbiochem, San Diego, CA, USA |
| Cisplatin | #232120 |  |
| Aldefluor detection kit | # 01700 | STEMCELL Technologies Inc, Vancouver, Canada |
| Cancer stem cell media | C-28070 | Promo cell, Heidelberg, Germany |
| Pacific Blue-Annexin V staining kit | #11 858 777 | Roche, Basel, Switzerland |
| IRDye 680 LT goat anti mouse IgG, red | 926-68020 | Li-Cor, Lincoln, NE, U.S.A. |
| IRDye 800CW Donkey anti-Mouse IgG, green | 926-32212 |  |
| IRDye 800 goat anti rabbit IgG, green, | 926-32211 |  |
| IRDye 680RD Donkey anti-Rabbit IgG, red | 926-68073 |  |

**Supplementary figures:**


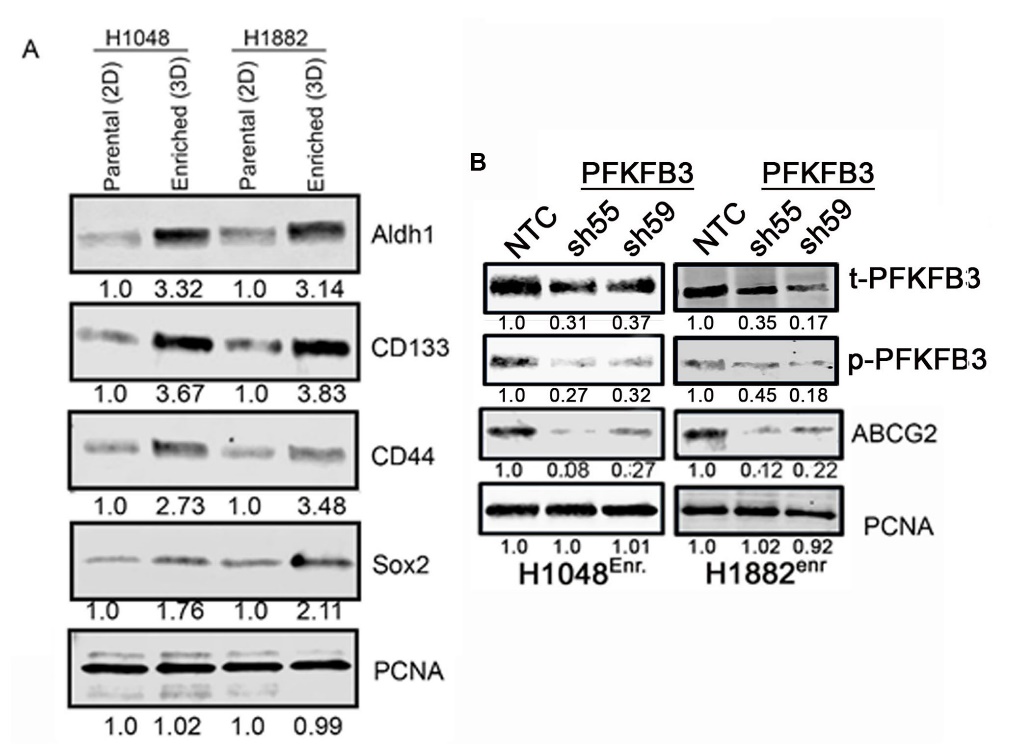


**Supplementary Figure 1** (**A**) The expression levels of Aldh1, CD133, CD44, and Sox2 in 3D tumor spheres and 2D adherent cells of H1048 and H1882 cells. (**B**) Level of total and phospho-PFKFB3 and ABCG2 expression in PFKFB3 KD cells of CSC enriched H1048 and H1882 spheroids.


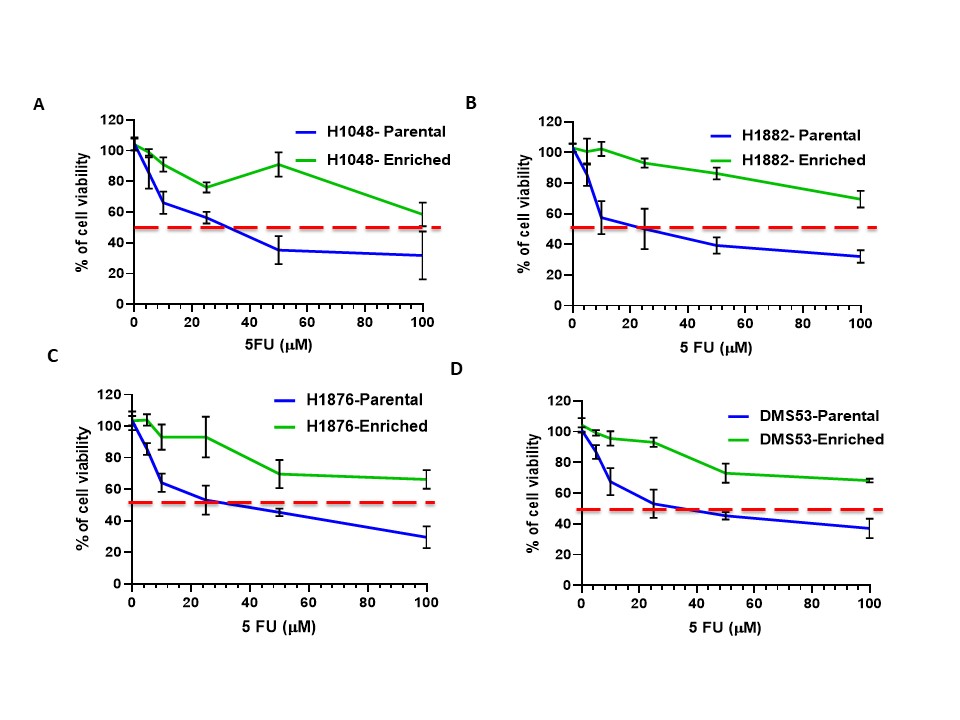


**Supplementary figure 2: Cytotoxicity of 5FU on SCLC cells and CSC enriched tumor spheroids**. Cell viability was measured following 24 h of 5FU treatment at indicated concentrations using MTT assay in both parental and CSC enriched tumor spheroids of (**A**) H1048, (**B**) H1882, (**C**) H1876 and (**D**) DMS53 cells. Data are expressed as the mean ± SD and experiments were conducted in triplicate and repeated independently three times (n = 3).


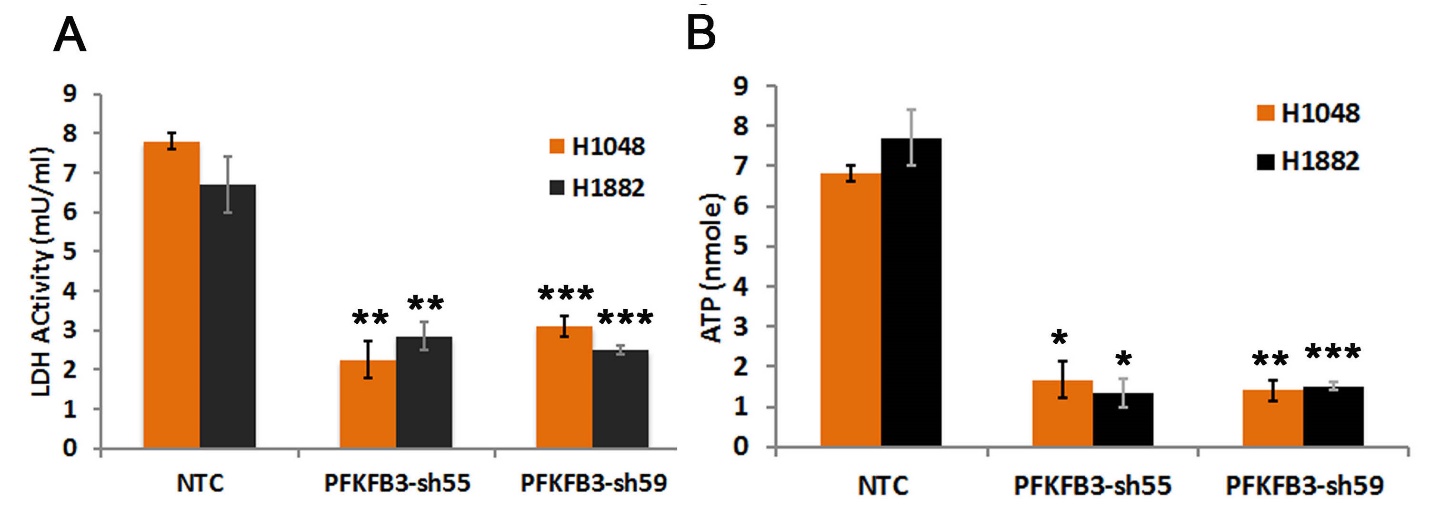
**Supplementary Figure 3: Genetic PFKFB3 inhibition reduces the LDH activity and ATP production of SCLC cells in vitro.** (**A**) Intracellular LDH activity and (**B**) ATP generation were measured in PFKFB3 knockdown of H1048 and H1882 tumor-spheres. *P < 0.05, **P < 0.01, ***P < 0.001.


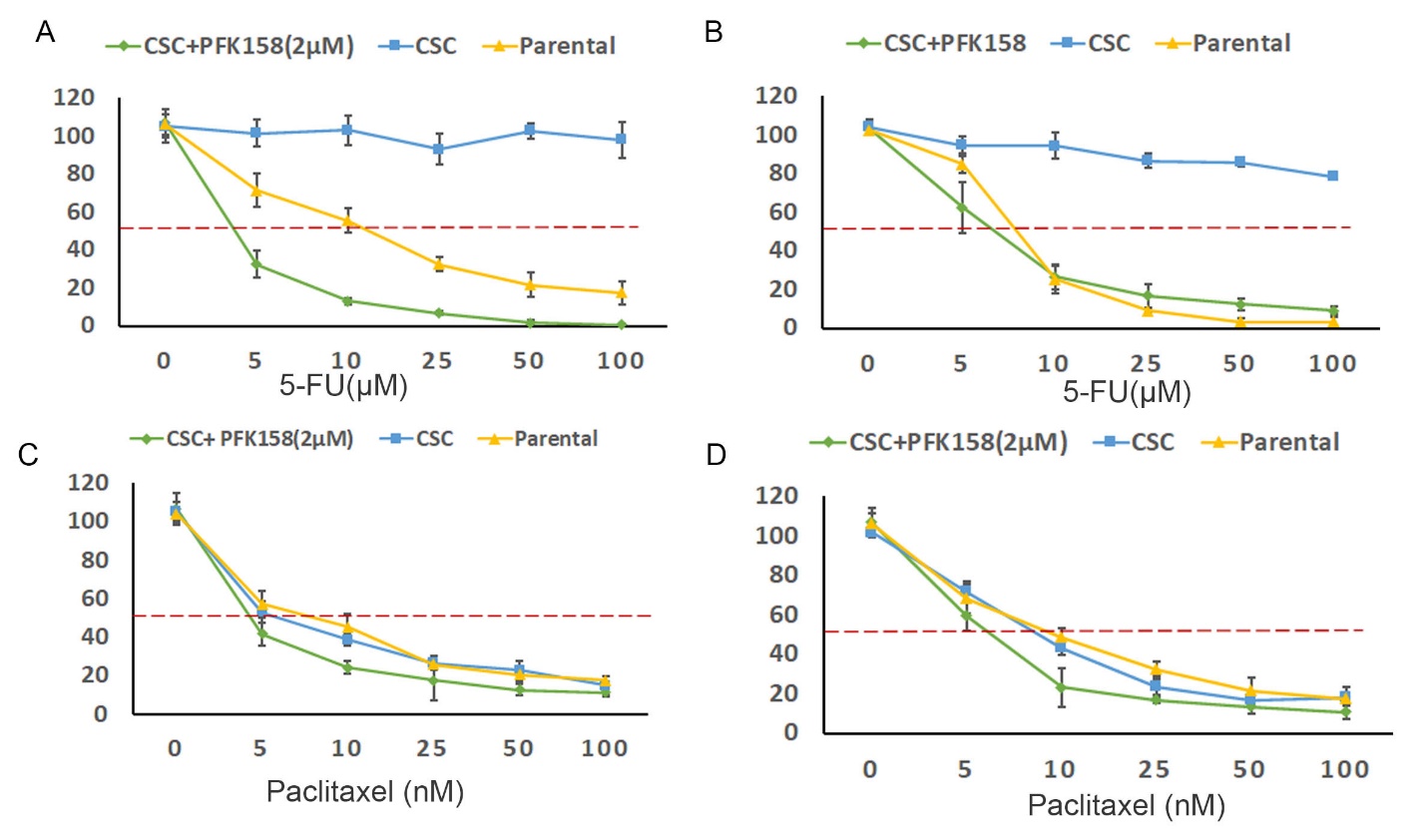


**Supplementary figure 4: PFK158 induces synergistic activity with ABCG2 substrate chemo therapeutic agents to inhibit CSC mediated proliferation of SCLC cells.** Both 2D (parental) and 3D-hangdrop (CSC) H1048 (**A**) and H1882 (**B**) cells were incubated with 5-Fluourouracil at specified range of concentrations in plus or minus PFK158 (2µM) for 24 h and cell viability was measured by Trypan blue dye exclusion assay. Both 2D and 3D type H1048 (**C**) and H1882 (**D**) cells were incubated with for non-ABCG2 substrate drug Paclitaxel (0-100nM) with or without PFK158 for 24 h and cell viability was measured. All experiments were performed in triplicate (n = 3)


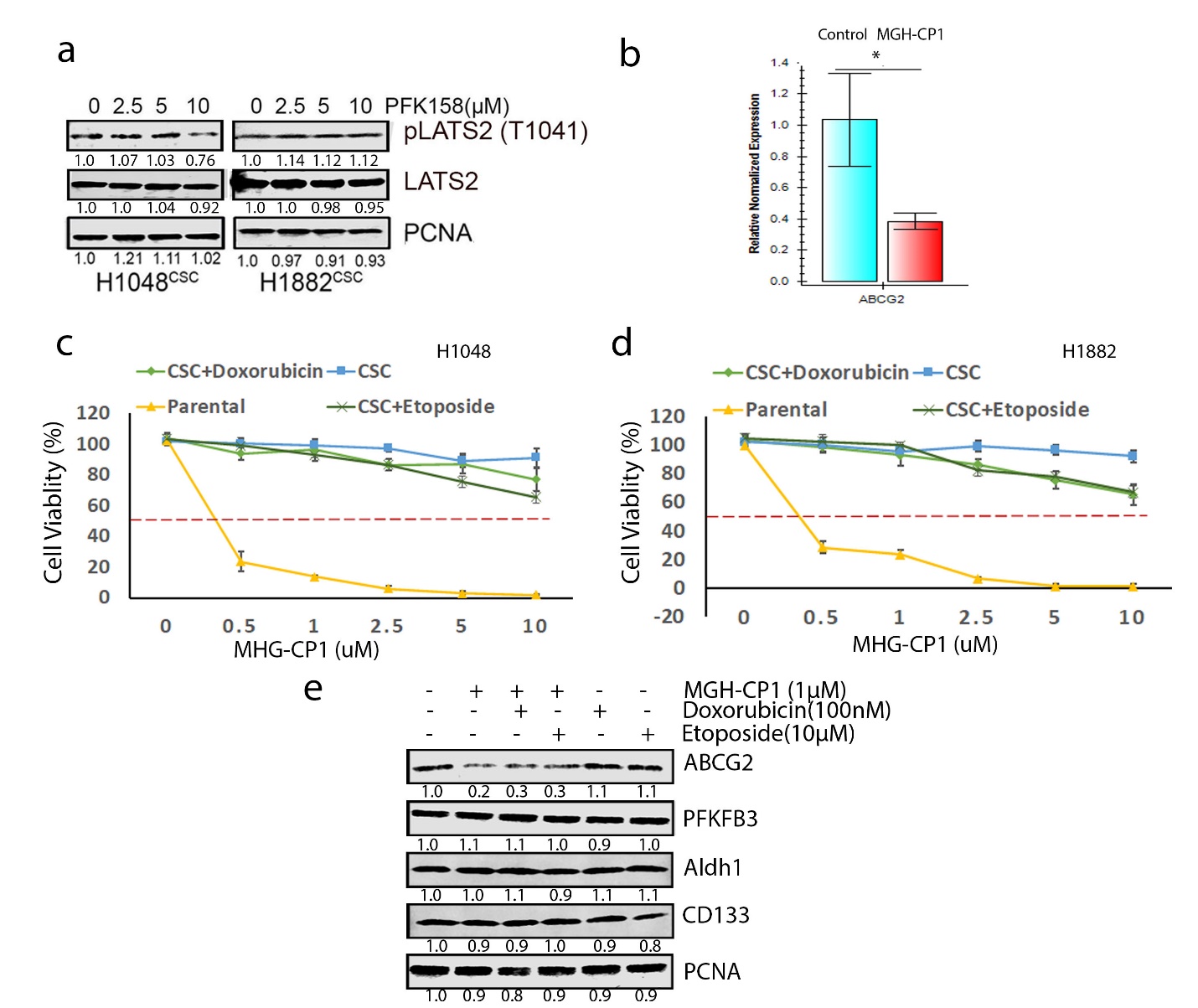


**Supplementary Figure 5: The role of PFKFB3 in EMT by PFK158 treatment or PFKFB3 knock down in SCLC xenograft model**. (**a**) Immunoblot analysis of pLATS2 and tLATS2 expressions from PFK158 treated SCLC-CSC cells. (b) mRNA level of ABCG2 in H1048^CSC^ cells after MGH-CP1(1µM) treatment for 24h. (c and d) Cytotoxicity of MGH-CP1in combination with or without Doxorubicin (100nM) and Etoposide (10 µM) in H1048 and H1882 cells respectively. (e) Western blot analysis of ABCG2, PFKFB3, Aldh1 and CD133 in H1048^CSC^ cells treated with MHG-CP1, Doxorubicin and Etoposide. *P < 0.05, **P < 0.01, ***P < 0.001.

**
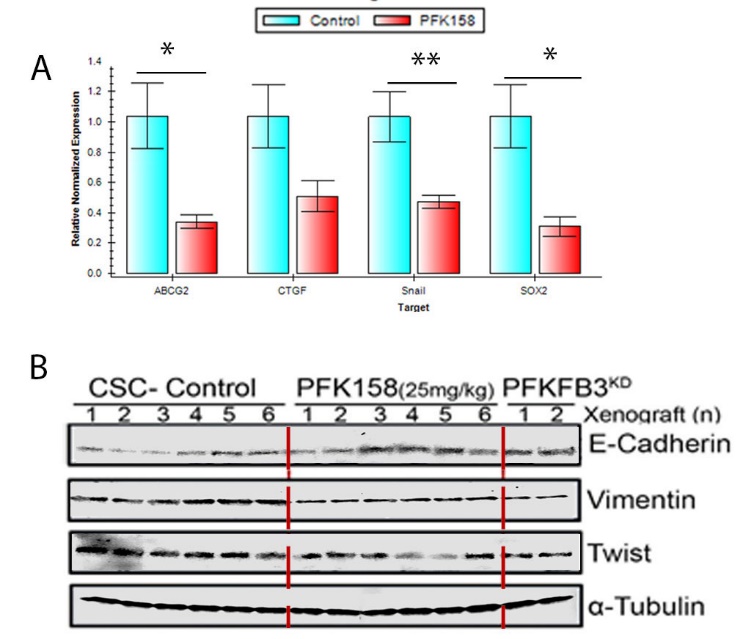
**

**Supplementary Figure 6: The role of PFKFB3 in EMT by PFK158 treatment or PFKFB3 knock down in SCLC xenograft model**. (A) Real Time-PCR analysis of YAP/TAZ target genes including ABCG2, CTGF, SOX2 and Snail from control and PFK158 treated SCLC xenografts. (B) Immunoblot analysis of epithelial mesenchymal transition markers expressions such as E-cadherin, vimentin, and twist from tumors of each group.
